# Microbiota and environmental health monitoring of mouse colonies by metagenomic shotgun sequencing

**DOI:** 10.1007/s11274-022-03469-0

**Published:** 2022-12-06

**Authors:** Laura Lupini, Cristian Bassi, Paola Guerriero, Marcello Raspa, Ferdinando Scavizzi, Silvia Sabbioni

**Affiliations:** 1 Department of Translational Medicine, University of Ferrara, 44121 Ferrara, Italy; 2 Present Address: Laboratorio per le tecnologie delle terapie avanzate (LTTA), University of Ferrara, 44121 Ferrara, Italy; 3 National Research Council (IBBC), CNR-Campus International Development, (EMMA- INFRAFRONTIER- IMPC), Monterotondo Scalo, Italy; 4 Department of Life Science and Biotechnology, University of Ferrara, Via Luigi Borsari 46, 44121 Ferrara, Italy

**Keywords:** Environmental health monitoring, Exhaust dust collection filters, ED filter, Exhaust Air dust (EAD) collection filters, Gut microbiota, Health surveillance, Laboratory mice, Metagenomics, Microbioma, Mouse colonies, NGS shotgun sequencing

## Abstract

Metagenomic next-generation sequencing (mNGS) allows the monitoring of microbiota composition of murine colonies employed for scientific purposes in a single test by assessing the composition of gut microbiome and the detection of pathogens from fecal pellets. In this study, we tested the potential use of mNGS for monitoring both microbiota composition and the presence of pathogens through Environmental Health Monitoring, by using exhaust dust collection filters derived from individually ventilated cages (IVC) systems.

mNGS analysis was performed on nucleic acids isolated from filters collecting air from the exhaust of: (1) cages with mice housed in a non-pathogen free facility; (2) animal-free cages with clean chow and bedding from the same facility; (3) cages housing mice from a specific-pathogen free (SPF) facility. mNGS results revealed correspondence between microbiome composition from fecal pellets and filter, including pathogenic bacteria (*Helicobacter hepaticus, Helicobacter typhlonius, Chlamydia muridarum, Rodentibacter pneumotropicus, Citrobacter rodentium*), intestinal protozoa (*Tritrichomonas muris, Spironucleus muris)* nematoda *(Aspiculuris tetraptera*) and eukaryotic parasites (*Myocoptes musculinus*), present in the colony. *Entamoeba muris* and *Syphacia obvelata* were detected in fecal pellets but not in filter. The animal free exhaust dust filter, exposed to clean cages (no mice) placed in the IVC after removal of all mice, exhibited the presence of the same pathogens due to contaminated connecting pipes, confirming the sensitivity of the approach. Conversely, the filter from SPF colony revealed the absence of pathogens.

The current use of exhaust dust collection filters in health surveillance requires multiple molecular tests to identify specific pathogens and does not provide information on the colony microbiome. This work provides the proof-of-principle that assaying exhaust dust collection filters by mNGS for microbiota monitoring of laboratory mice is feasible. In its daily application, results suggest the usefulness of the test in SPF facilities, where pathogenic micro-organisms are expected to be absent. mNGS analysis of exhaust dust collection filters allows the analysis of multiple cages, reducing the number of tests required for pathogen detection and corresponding costs, and avoiding the use of sentinel mice.

**Supplementary Information:**

The online version contains supplementary material available at 10.1007/s11274-022-03469-0.

## Introduction

Every phase of biomedical research that involves breeding and housing of laboratory animals, requires to operate in conditions that guarantee animals welfare and psychophysical health, in compliance with current regulations. Health monitoring of animals, usually rodents, is necessary in this regard, in order to ensure not only the maintenance of health status but also high ethical and scientific standards (Buchheister and Bleich 2021; Miller and Brielmeier, 2018).

Microbiological control has been traditionally aimed at detecting pathogenic agents, listed in the Federation of European Laboratory Animal Science Associations (FELASA) guidelines, potentially present in the animal colony. To this aim, sentinel animals, exposed to dirty bedding from other cages of the colony, are considered representative of the health status of the whole colony (Compton et al., 2004a; Lipman and Homberger, 2003). Subsequent tests on the sentinels consist mainly of serological and tissue analyses, based on microscopy and culture, usually involving sacrifice of the animal. However, employment of bedding sentinels in health monitoring programs cannot be totally justified on the basis of infectious agent transfer efficiency, which has been shown to be variable and generally insufficient (de Bruin et al., 2016). Moreover, ethical and regulatory reasons require that animals should not be used unless absolutely necessary, in compliance with the 3R (Replacement, Reduction, Refinement) guidelines (WMS and Russel 1959).

An alternative monitoring approach is the microbiological monitoring of the colony directly on colony animals or from particle samples on IVCs rack exhaust dust environmental collection filters, by molecular methods such as PCR or RT-PCR to diagnose bacterial, viral, fungal infections, reducing or completely eliminating the use of sentinels, in compliance with the 3R reduction principle (Korner et al., 2019; Mahabir et al., 2019; Manuel et al., 2017) (Miller and Brielmeier, 2018) (Pettan-Brewer et al., 2020) (Zorn et al., 2017). PCR on nucleic acids extracted from filters has proven to be more sensitive and effective than the use of sentinels for the detection of specific pathogens such as *Murine norovirus* (MNV) (Zorn et al., 2017), *Mouse hepatitis virus* (MHV) (O’Connell et al., 2021), *Sendai virus* (Compton et al., 2004b), Murine Astrovirus (Korner et al., 2019), *Pasteurella pneumotropica* (Miller et al., 2016), *Helicobacter* spp. (Mailhiot et al., 2020), *Lactate dehydrogenase elevating virus* (LDV) (Luchins et al., 2020b), *Pneumocystis murina* (Miller and Brielmeier, 2018), fur mites (Hanson et al., 2021) *(*Gerwin et al., 2017) *(*Korner et al., 2019) Protozoa and pinworm (Kapoor et al., 2017) (Dubelko et al. 2018) (Bauer et al., 2016). This rodent-free approach is ethical reducing animals and animal manipulations. Finally, environmental health-monitoring programs were found to be qualitatively superior (Mailhiot et al., 2020) (Kimie Niimi,^2018^) and less expensive than sentinel based programs, decreasing also the time spent by the staff on heath-monitoring activities (Luchins et al., 2020a).

PCR has several advantages such as speed, sensitivity, specificity, thanks to primers designed specifically for amplification based on available knowledge, but it is not without limits. The method allows only the microorganisms (usually pathogens) sought to be identified and does not provide information on the composition of the whole animal microbiota, which is essential for the correct development of the host organism, for host health and for response to therapies (Lazar et al., 2018; Lozupone et al., 2012) (Tang et al., 2019) (Kau et al., 2011) (Ley et al., 2006) (Arpaia et al., 2013) (Smith et al., 2013) (Furusawa et al., 2013) (Levy et al., 2017) (Arentsen et al., 2015) (Liu et al., 2019) (Antonini et al., 2019) (Caspani and Swann, 2019) (Fung et al., 2017; Sampson and Mazmanian, 2015) (Sharon et al., 2016) (Vicentini et al., 2021) (Needham et al., 2022; Yu et al., 2019) (Matson et al., 2021) (Pernigoni et al., 2021). Also, for rapidly evolving agents, such as RNA viruses, single nucleotide changes in the primer regions can potentially lead to false negative, as the agent might be present but the target not amplified (Compton, 2020).

An approach that can go beyond the above-mentioned ethical and technical limits and could be used for colony health monitoring is metagenomic next generation sequencing (mNGS). mNGS can provide sequencing of all the nucleic acids present in the sample under analysis, of host and of microbial origin. Unlike NGS methods based on sequencing of 16 S rRNAs, mNGS is not limited to bacterial sequences detection only, but it allows detection of viruses, fungi, and parasites. Moreover, unlike single-strain PCR testing, it allows a comprehensive and quantitative assessment of the sample microbiota (Salipante et al., 2014), enables species and strain identification (Salipante et al., 2015) and discovery of new organisms (Chiu, 2013). The effectiveness of the mNGS approach was demonstrated in a study that aimed to monitor the intestinal microbiota and detect pathogens in murine colonies directly from fecal samples and avoiding sentinels (Scavizzi et al., 2021).

Here we investigated the use of mNGS for microbiota and microbiological monitoring of the colonies through analysis of particles present on environmental collection filters in IVCs racks.

## Materials and methods

***Mouse strains and housing facility***. C57BL/6NTacCnrm (B6N) mice, between 8 and 12 weeks of age were housed and bred in facilities accredited by the Italian Ministry of Health in accordance with the Italian legislation Dlgs. 26/2014 and European directive 63/2010. Analyses of fecal pellets were previously published (Scavizzi et al., 2021). For the SPF facility: mice (n = 5 mice per cage, total 20 cages) were housed in sealsafe greenline rack in individually ventilated cages (Tecniplast, Gazzada, Italy) in positive pressure, under a 12:12 light: dark cycle with 70 air changes per hour (ACH) with autoclaved rodent chow (4RFN and EMMA 23, Mucedola, Settimo Milanese, Milano, Italy) and autoclaved tap water ad libitum and bedding (Scobis one, Mucedola, Settimo Milanese, Milano, Italy). Autoclaved enrichments and nesting material are present in each cage. SPF is a confined environment with dedicated personnel where only breeding colonies are maintained. Materials are autoclaved and personnel enter after a wet shower and a complete change with sterile clothes. All mice were monitored and found negative for the pathogen of the FELASA recommendations list. For the non SPF facility: mice (n = 5 mice per cage, total 11 cages) were housed in individually ventilated cages (Tecniplast, Gazzada, Italy) in positive pressure, under a 12:12 light: dark cycle with 70 air changes per hour (ACH) with rodent chow (Teklad global diet 2018, Envigo, San Pietro al Natisone, Udine, Italy) and tap water ad libitum and bedding (Lignocel Bk 8/15, Envigo, San Pietro al Natisone, Udine, Italy). Autoclaved enrichments (rodent polycarbonate tunnel tubes) and nesting material are present in each cage. Personnel enter after wearing disposable clothes and shoe covers, gloves, surgical masks and bonnets. All non SPF mice were routinely monitored to assess the health status by standard and molecular methods (PCR, mNGS) for the pathogens listed in the FELASA recommendation list and were found positive for the following pathogens: *Tritricomonas muris*, *Entamoeba muris, Spironucleus muris, Syphacia obvelata, Aspiculuris tetraptera*, *Myocoptes musculinus*; *Helicobacter spp. Chlamydia muridarum, Streptococcus pyogenes Rodentibacter pneumotropicus, Citrobacter rodentium, Staphylococcus aureus* as previously described (Scavizzi et al., 2021). Animal–free control: all cages containing animals were removed from the IVC system in the non SPF room and replaced with clean cages without animals, containing only clean rodent chow (Teklad global diet 2018, Envigo, San Pietro al Natisone, Udine, Italy) and bedding (Lignocel Bk 8/15, Envigo, San Pietro al Natisone, Udine, Italy) and no mice. The IVC rack was not cleaned before introducing the animal free cages.

***Exhaust dust (ED) collection filters***. Exhaust dust collection filters “Interceptor” are a patented system from Tecniplast (Tecniplast, Gazzada, Italy). The “Interceptor” filter aims to collect air and dust moving from cages to the exhaust filtration area of the air handler unit (AHU). To ensure that most of the particles in the exhaust air came into contact with the filter, the filter was inserted directly at the end of the exhaust air hose, immediately before the exhaust filtration area of the AHU.

An exhaust dust collection filter (TEST filter) was placed at the end of the exhaust air hose, which collects air and dust from 11 individually ventilated cages (IVC) housing mice (n = 5 mice per cage) of a non SPF facility. The remaining cages in the IVC were clean but empty (no bedding, no chow, no mice). Although it has been shown that already after 15 days exhaust air filter analysis gives positive PCR results, when exposed to air dust of infected mice (Miller et al., 2016), an increased exposure time (30 days) was chosen to facilitate identification of the microbial genomes composing the microbiome. After 30 days, the TEST filter was collected, DNA and RNA purified and analyzed by mNGS. Sequence data displayed 2.7 × 10^6^ high-quality filtered reads, of which 64,411 reads (2.4% of total) matched to microorganism genomes.

An exhaust dust collection filter (SPF filter) was placed for 30 days in a SPF IVC system housing 20 ventilated cages (n = 5 mice per cage) and analyzed by mNGS. Sequence data displayed 5 × 10^6^ high-quality filtered reads, of which 87,801 reads (1.8% of total) matched to microorganism genomes.

***Purification of nucleic acids.*** Environmental collection filters (Interceptor, Tecniplast), placed at the end of the exaust air collection tube, in the individually ventilated cages system (IVC) system, were collected, sterilely cut and transferred into a sterile, DNA-free Eppendorf tube. Lysis Buffer (MC501C, Promega) was added and microbial nucleic acids were isolated using the Promega Maxwell® RSC system (AS4500, Promega), following the manufacturer’s instructions. As for the fecal pellets, for non SPF mice: 5 pellets per cage (11 cages) were collected;for SPF mice: 5 pellets per cage (5 cages) were collected. Separated pools were generated from fecal pellets deriving from each cage. DNA/RNA extraction was carried out on each pool/cage separately. As for the clean bedding and chow sample, DNA was purified from a pulverized sample of chow and bedding taken from a microisolator cage (no animals) placed in the non SPF IVC rack for 4 weeks, and was used for library preparation and sequencing (Scavizzi et al., 2021).

***Library preparation and sequencing.*** Nucleic acids from ED filters were used for library preparation and sequencing as previously described (Scavizzi et al., 2021). Briefly, nucleic acids were retro-transcribed to convert RNA to cDNA before library preparation. RNA was retro-transcribed using the following reagents: RevertAid H Minus Reverse Transcriptase (200 U/µL) (EP0451, Thermo Scientific); RNaseOUT™ Recombinant Ribonuclease Inhibitor (10,777,019, Invitrogen); Random Primers (48,190,011, Invitrogen); 10mM dNTP Mix (P/N y02256, Invitrogen), DTT 0.1mM (P/N y00147, Thermo Scientific). After incubation of RNA with Random Primers for 5 min at 70 °C, the remaining reagents were added and cDNA was synthetized at 37 °C for 1 h, followed by a 5 min-incubation at 94 °C.

Libraries were prepared using NEBNext Fast DNA Fragmentation & Library Prep Set for Ion Torrent (New England Biolabs # E6285L). Briefly, 50 ng of DNA were fragmented, end-repaired and Ion Torrent specific-motifs from Ion Xpress Barcode adapters (Thermo Fisher # 4,471,250) were ligated to both ends of DNA fragments. Agencourt AMPure XP magnetic beads (Beckman Coulter #A63881) were used to perform a size-selection, allowing to select 200 bp DNA fragments, that were successively amplified (9 cycles). Finally, libraries were cleaned-up through Agencourt AMPure XP beads and quantified with Agilent High Sensitivity DNA kit (Agilent # 5067 − 4626), using the Bioanalyzer 2100 instrument. No adapter contamination or primer-dimers was detected by Bioanalyzer tracing. Libraries were pooled and subjected to template preparation and sequencing, according to Ion 540™ Kit-OT2 protocol (Thermo Fisher # A27753). Sequencing was performed on an Ion 540 chip (Thermo Fisher #A27765), using the Ion GeneStudio S5 System (Thermo Fisher), which yielded 1 gigabases of high-quality data with an average of 5.9 × 10 ^6^ reads per sample (range: 2,775,874 -9,944,212 reads per sample).

***Bioinformatics and statistical analysis.*** Bioinformatics and statistical analysis were performed as previously described (Scavizzi et al., 2021). Briefly, quality control with FASTQC has been performed and nucleotides with a quality score less than 20 (MAPQ < 20) have been trimmed. Reads shorter than 100 nucleotides were filtered out from raw FASTQ files, using PRINSEQ-lite 0.20.4 (prinseq-lite.pl -min_len 100) (Schmieder and Edwards, 2011). Reads matching the mouse genome were removed using bowtie2 v.2.3.2 (bowtie2 -p 15 -x mm10_reference_genome) (Langmead and Salzberg, 2012) and samtools 1.4 (samtools view -f4 and samtools fastq) (Li et al., 2009). The remaining reads were used to perform taxonomy calling at genus and species levels, using Kraken 2 (kraken2 --db kraken_reference_db --threads 20 --confidence 0.5 --report sample.kreport --report-zero-counts) (Wood and Salzberg, 2014), Bracken (bracken -d kraken_reference_db -i sample.kreport -o sample_species.braken.tmp” -r 150 -l S (also -l G, -l P, -l C, -l F)) (Lu et al., 2017), and a database consisting of all the complete and draft genome sequences in GenBank Release 232 of archaea, bacteria, fungi, protozoa, virus and invertebrate endo- and ecto-parasites of mice (*Acantocephala*, *Annelida*, *Helminths* and *Nematoda*). Kraken2 was run with default parameters but with a confidence score set to 0.5 to increase the precision. Each classified sequence (read) was attributed to its last known taxon (LKT). Genus and species with zero counts in all the samples were removed. The R programming language (version 3.5.0) was used to assemble in a single table all metagenomic data. Analyses and data plot were performed with Prism version 6.0 (GraphPad Software) unless otherwise stated.

### Nucleotide sequences accession number

The data that support the findings of this study are available through the European Nucleotide Archive ENA under accession number PRJEB55812.

## Results

### Gut microbiome composition of non SPF mice is reliably assessed from exhaust dust collection filters by metagenomic shotgun sequencing

**Fig. 1:**
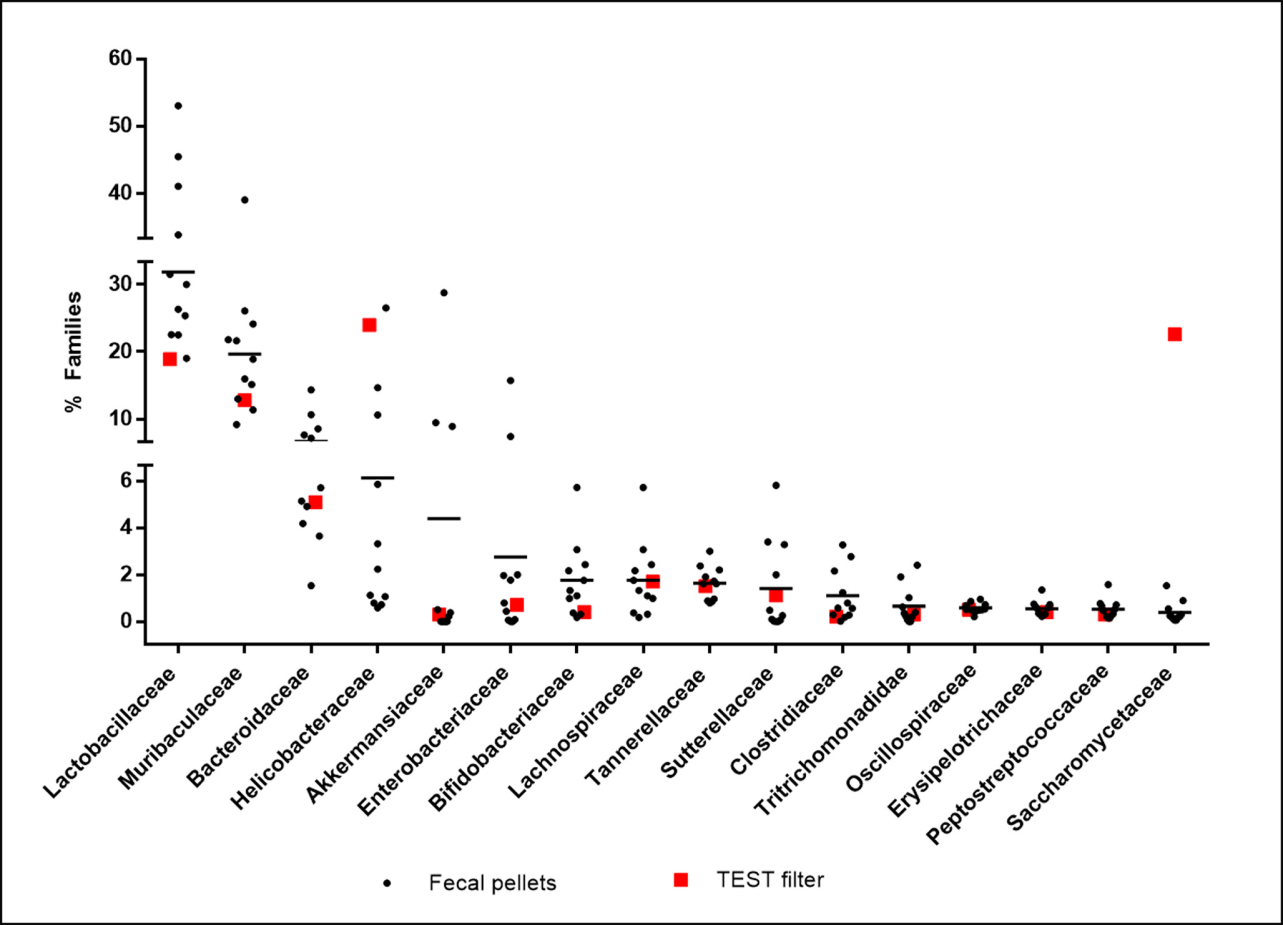
Comparison of mouse gut microbiome assessed from ED filter and from fecal pellets. Graph represents 16 microbial families (x axis) detected (average read counts ≥ 0.2**%**)on the ED filter and in mice fecal pellets collected from 11 cages of the non SPF facility. For each family: percentages of the microbial families in fecal pellets (black dots) and in ED filter (red squares) are shown. Mean is represented by a black line

The analysis of microorganism sequencing data revealed the composition of the microbiome present on filter, which was compared to microbiome of fecal pellets collected from the 11 cages present in the same IVC system (Additional file1). The relative abundance of 16 microbial families (with average read counts ≥ 0,2% in both types of samples) was found to be similar on filter and in fecal pellets (Fig. 1).

At the species level, 40 species identified in fecal pellet with a mean read count above 1000, were also correctly identified in the ED filter (ED detection = 100%); among the 31 species identified in fecal pellet with a mean read count range < 1000 and > 200, 23 were also correctly identified in the ED filter (ED detection = 74%); among the 81 species identified in fecal pellet with a mean read count range < 200, 28 were also correctly identified in the ED filter (ED detection = 35%). For species with a mean read count above 1000 there is no statistically significant difference between fecal pellets and filter. For species with a mean read count range < 1000, pellets are statistically more significant than the filter (Table 1**)**.

**Table 1:** Species identified in ED filter vs. fecal pellets by mNGS

Average read counts of species identified in fecal pellets	Number of species identified in fecal pellets	Number of species identified in filter	Concordance between fecal samples and filter	P value*
> 10,000	17	17	100%	1
range > 1000 and <10,000	23	23	100%	1
range > 200 and < 1000	31	23	74%	0,005
< 200	81	28	36%	< 0,001

• P value calculated by Fisher Exact test

In total, ED filter analysis made it possible to correctly identify 91 species out of a total of 152 identified in the fecal samples (60%). These results provide the proof of principle that the ED filter allows to trace the composition of the gut microbiome of the mice housed in a IVC system.

Among microorganisms that differ between ED filter and fecal pellets, S*accharomycetaceae* family is the most notable: it makes up < 1% of the counts detected in feces, it accounts for almost 23% of the counts detected on the filter. Since NGS analysis can also detect families that are constituents of plant microbiome attributable to the chow and bedding present in the cages, S*accharomycetaceae* are therefore identified on the filter due to the dust deposition from these materials. Six additional families (*Erwiniaceae, Pseudomonadaceae, Pasteurellaceae, Nectriaceae, Cephalobidae and Moraxellaceae*) were identified in the ED collection filter but were not detected in mouse fecal pellets. Also these families are attributable to the chow and bedding present in the cages and whose dust is collected by ED filter (Table 2**)**.

**Table 2:** Comparison between family percentage in non SPF mouse fecal samples, ED TEST filter, and clean bedding and chow

Families	Mouse fecal pellets (average percentage)	ED TEST filter	Clean bedding and chow
*Lactobacillaceae*	39.9%	18.9%	3.7%
*Muribaculaceae*	24.6%	12.8%	0.2%
*Bacteroidaceae*	8.4%	5.1%	nd (0.05%)
*Helicobacteraceae*	7.7%	24%	Nd
*Akkermansiaceae*	5.5%	0.3%	Nd
*Enterobacteriaceae*	3.4%	0.7%	0.15%
*Bifidobacteriaceae*	2.2%	0.4%	Nd
*Lachnospiraceae*	2.1%	1.7%	Nd
*Tannerellaceae*	2.0%	1.5%	Nd
*Sutterellaceae*	1.7%	1.1%	Nd
*Clostridiaceae*	1.4%	0.2%	0.2%
*Tritrichomonadidae*	0.8%	0.3%	Nd
*Oscillospiraceae*	0.7%	0.5%	nd (0.03%)
*Erysipelotrichaceae*	0.7%	0.4%	Nd
*Peptostreptococcaceae*	0.7%	0.3%	Nd
*Saccharomycetaceae*	0.5%	22.6%	88.9%
*Erwiniaceae*	Nd	0.6%	0.9%
*Pseudomonadaceae*	nd (0.05%)	0.5%	0.4%
*Pasteurellaceae*	nd (0.01%)	0.4%	0.1%
*Nectriaceae*	Nd	1.7%	1.3%
*Cephalobidae*	Nd	nd	2.5%
*Moraxellaceae*	Nd	1.4%	0.2%

In summary, ED filters can reveal both mice and bedding microbiome composition.

### Pathogens are detected from exhaust air dust collection filters by metagenomic shotgun sequencing

**Fig. 2:**
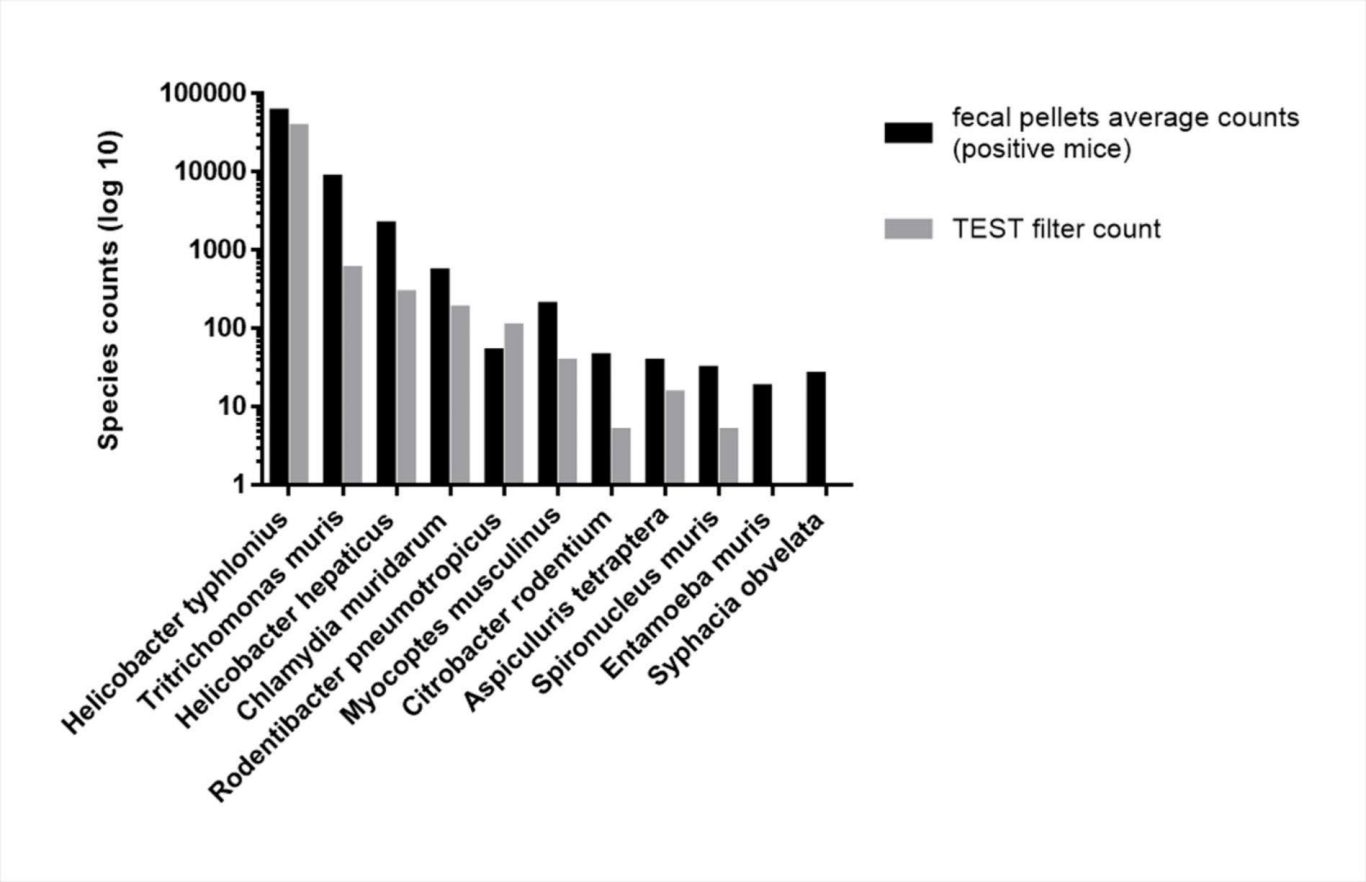
Pathogens assessed from fecal pellets and from ED filter. Graph represents pathogens species (x axis) detected by mNGS analysis. For each species: log 10 of average read counts of positive mice and read counts in ED filter form the non SPF facility are shown. Among the eleven species identified in at least one sample of fecal pellets, nine were also detected in ED filter. Two species, *Entamoeba muris* and *Syphacia obvelata*, whose read counts in fecal pellets were < 30, were not detected on ED filter

The mNGS analysis of the ED filter revealed the presence of 9 pathogenic species, belonging to bacteria (*Helicobacter hepaticus, Helicobacter typhlonius, Chlamydia muridarum, Rodentibacter pneumotropicus, Citrobacter rodentium*), intestinal protozoa (*Tritrichomonas muris, Spironucleus muris)* nematoda *(Aspiculuris tetraptera*) and eukaryotic parasites (*Myocoptes musculinus*). mNGS results from the ED filter were in agreement with those obtained directly from the fecal pellets and there are no statistically significant differences between the results of the pellet fecal and filter (p value = 1 according to Fisher Exact test). All the pathogenic species were detected on the air dust collection filter with a couple of exceptions, namely the intestinal protozoa *Entamoeba muris* and nematoda *Syphacia obvelata*, which presented an average read count in fecal pellets less than 30, respectively 18 and 26 reads (Table 3 and Fig. 2**)** and were found in the feces but not in the ED filter.

**Table 3:** Pathogen species identified in fecal pellets and in EAD collection filters by mNGS

Superkingdom	Species ID	Species	Number of positive cages/total	average read counts fecal pellets *	ED filter counts
Bacteria	76,936	*Helicobacter typhlonius*	11/11	59,213	37,780
Endo-and ecto parasite	5726	*Tritrichomonas muris*	10/11	8502	580
Bacteria	32,025	*Helicobacter hepaticus*	11/11	2173	285
Bacteria	83,560	*Chlamydia muridarum*	5/11	546	182
Bacteria	758	*Pasteurella pneumotropica*	6/11	52	107
Endo-and ecto parasite	1,046,713	*Myocoptes musculinus*	4/11	202	38
Bacteria	67,825	*Citrobacter rodentium*	1/11	45	5
Pinworm nematode	451,377	*Aspiculuris tetraptera*	5/11	38	15
Endo-and ecto parasite	39,710	*Spironucleus muris*	4/11	31	5
Endo-and ecto parasite	545,931	*Entamoeba muris*	4/11	18	0
Pinworm nematode	412,127	*Syphacia obvelata*	1/11	26	0

* Average counts from positive pellets only

To provide an animal-free control, all cages containing animals were removed from the IVC system and replaced with clean cages without animals, with clean chow and bedding and no mice. A new ED (animal-free filter) was placed for 30 days in the same IVC system and analyzed by mNGS. Sequence data displayed 5 × 10^6^ high-quality filtered reads, of which 287,285 reads (5.7% of total) matched to microorganism genomes.

**Fig. 3:**
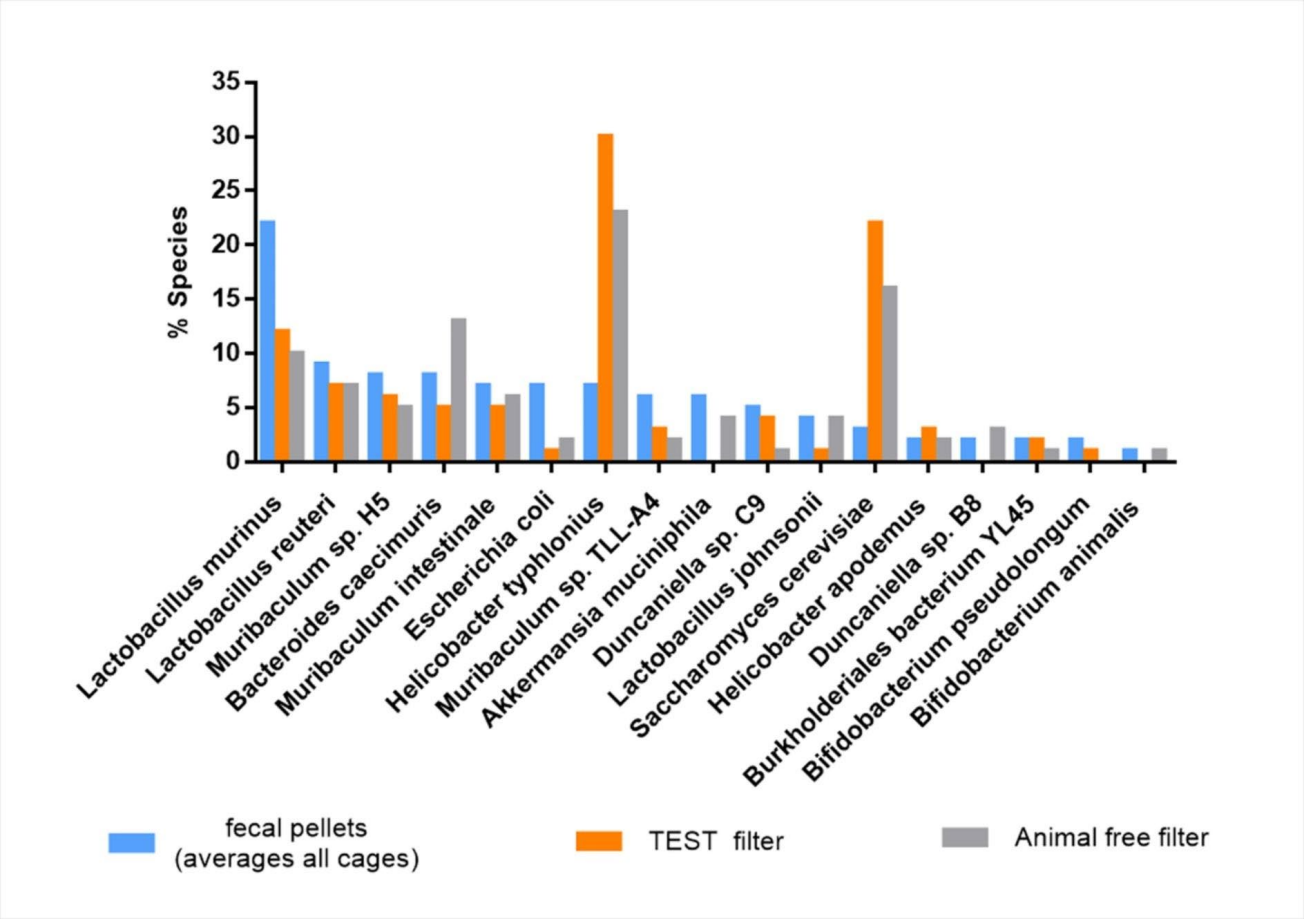
Comparison between species % assessed by mNGS: from fecal pellets (average counts > 10,000) blue bars; from ED TEST filter orange bars; from ED animal-free filter grey bars. In the non SPF facility, 100% of the species detected in the ED TEST filter were also present ED animal-free filter, despite the cages being empty, showing that the filter is representative not only of what the IVC rack contains but also of what it contained, due to the presence of dust residues from the housing cages and from the collector tubes

As expected, the animal-free filter displayed several specific taxa of plant microbiome due to dust derived from chow and bedding. However, all of the species (with average counts in fecal pellets > 10,000) detected in the TEST filter were also present in the animal-free filter, despite the absence of mice, showing that the filter is representative not only of what the rack contains but also of what it contained, likely due to the presence of dust residues from the previous housing cages from contaminated collector tubes of the IVC rack (Fig. 3).

The ability of the filter to detect pathogens present not only in a group of cages, but in the connecting pipes of the IVC system itself, suggests its possible use in health surveillance, especially of SPF facilities, where the presence of pathogenic micro-organisms is excluded *a priori*. To this end, an environmental filter from a SPF facility (SPF filter) was analyzed, revealingthe absence of pathogens on the filter and confirming what observed by mNGS directly on fecal samples of the SPF mice (Additional file2). No pathogens were found in the filter nor in the fecal pellets, confirming the correct microbiological level /status of the SPF colony.

Regarding the microbiome composition of the SPF mice, the SPF filter correctly identified the 8 families (*Muribaculaceae, Lactobacillaceae, Bacteroidaceae, Lachnospiraceae, Bifidobacteriaceae, Erysipelotrichaceae, Oscillospiraceae, Peptostreptococcaceae*), that make up 98% of the families identified in fecal pellets and that are present with an average read counts 0,2% both on the filter and in the feces. (Additional file 4).

## Discussion

A shotgun metagenomics NGS (mNGS) approach was performed to investigate mouse microbiome and the possible presence of pathogens in enviromental collection filters derived from IVC housing murine colonies. We have previously demonstrated that mNGS technology allows to detect any microorganism present mainly but not only in the gut of mice directly from fecal samples, providing a detailed description of the sample’s commensal microbiota and avoiding the need to carry out multiple tests for the detection of individual pathogens (bacteria, viruses, fungi, and parasites) (Scavizzi et al., 2021). In this previous work it has been shown that technical reproducibility is very high with duplicates showing a correlation coefficient > 99%. The purpose of the present work was to determine the composition of the intestinal microbiota and to identify the presence of pathogens directly from ED filter by mNGS. The present study demonstrates that this goal can be achieved. The dust was analyzed after 30 days of exposure, even though manufacturer indicates 90 days, to have a more rapid indication if this approach would be similarly feasible and accurate. Moreover, it has been shown that already after 15 days, exhaust air filter analysis gives positive PCR results, when exposed to air dust of infected mice (Miller et al., 2016) The dust collected by the ED filter summarized the composition of microbiome assessed directly in fecal pellets, allowing the identification of the most abundant microbial families present in both type of samples with (average read counts ≥ 0,2%). At the species level, 91 species were correctly identified by the ED filter mNGS test, out of a total of 152 identified in the fecal samples. Overall, 60% of species were identified by the ED mNGS test compared to fecal samples, while 40% of species were not identified by the ED mNGS test. However, bedding microbiome composition was additionally identified by the ED mNGS test.

The results provide the proof of principle that the ED filter allows to trace the composition of the gut microbiome of the mice housed in a IVC system. The presence of pathogenic bacteria (*Helicobacter (*Taylor et al., 2007) *hepaticus, Helicobacter typhlonius, Chlamydia muridarum, Rodentibacter pneumotropicus (*Benga et al., 2018), *Citrobacter rodentium, Staphylococcus aureus*), intestinal protozoa (*Tritrichomonas muris, Spironucleus muris)* nematoda *(Aspiculuris tetraptera*,) and eukaryotic parasites (*Myocoptes musculinus*) was demonstrated by environmental filter mNGS test. All pathogens identified in the feces and presenting an average read count > 30 were represented in the filter. The filter mNGS test failed to detect two of eleven pathogens identified in fecal samples, namely *Entamoeba muris* and *Syphacia obvelata;* these species were found in the feces with average count less than 30 reads, which therefore seems to indicate the limit of sensitivity of the filter mNGS test. This limit of sensitivity could be probably reduced or overcome exposing the filter for a longer period of time (e.g. 90 days) as described (Mailhiot et al., 2020) (Gerwin et al., 2017) and improving sequencing depth. It is worth mentioning that false negative PCR results from environmental samples were already observed, in particular with pinworms (Kapoor et al., 2017). No false-positive results were produced by the ED mNGS test, since all pathogens identified from the filter have also been identified in the feces of the animals both by NGS and by standard molecular testing (PCR) (Scavizzi et al., 2021), which is currently considered standard method for the detection of mouse pathogens directly on colony animals (Miller and Brielmeier, 2018).

We observed that, mNGS test performed on a filter exposed to clean chow and bedding revealed the persistence of pathogens likely derived from the contaminated collection pipes of the IVC system after the removal of mice from the rack. This result, already described by Pettan-Brewer (Pettan-Brewer et al., 2020) and Miller (Miller et al., 2016) confirms, on the one hand, the need to periodically clean and sanitize the IVC system and the collector tubes. On the other hand, it suggests the possibility of using the mNGS ED test for the health surveillance of SPF colonies where the possible occurrence of pathogens could be easily monitored from filter analysis. In fact, the ED mNGS test of a SPF IVC rack revealed the absence of pathogens in the colony. To this end, the ED mNGS test could provide several advantages in pathogens surveillance of SPF colonies, namely (1) the increased sensitivity of ED monitoring compared to traditional sentinel-based methods, eliminating the need of sentinels dedicated to health monitoring, in accordance with the 3R principle; (2) the possibility to avoid the sampling of feces from individual cages with a reduction of the costs of purification of nucleic acids and the costs related to time spent by the staff on health-monitoring activities; (3) the completeness of the information that mNGS analysis offers compared to the partial picture that can be obtained with ED PCR assays, replacing a variety of specific tests and allowing the characterization of sample microbiota; (4) compared with the costs associated with multiple single PCR testing, costs associated with this technology are now comparable, if not advantageous.

There is a debate if all pathogens can be similarly detected by enviromental collection filters, and this depends on the route and the duration of transmission of the infectious agents (Compton et al., 2004a). Agents that are transmitted by animal-animal contact or by fecal-oral route like Mouse *Parvovirus* (Bauer et al., 2016) or Mouse *Rotavirus* (Compton et al., 2004b) could be detected less efficiently by environmental air monitoring, while, on the contrary, respiratory virus like *Sendai* are perfectly identified on enviromental filters (Compton et al., 2004b). Recently, however, a different environmental approach has been demonstrated sensitive for the detection of MPV (O’Connell et al., 2021). Many publications describe that environmental EAD analysis is qualitatively superior and economically convenient compared to classical sentinel system (Miller and Brielmeier, 2018) (Luchins et al., 2020a) (Mahabir et al., 2019) (Mailhiot et al., 2020) (Manuel et al., 2016) (Manuel et al., 2017) (Miller and Brielmeier, 2018; Miller et al., 2016) (Pettan-Brewer et al., 2020) (Zorn et al., 2017). To extend the general significance of the results, our future work will analyze filters from different facilities and with different exposure times in order to verify the variability of the system. Although in this case viruses were not present in the colony and therefore not detectable on filters, the possibility of detecting viruses in pellet fecal by mNGS had been demonstrated (Scavizzi et al., 2021). Even if this work couldn’t prove positive detection of all possible microorganisms, since not present in the colony, it provides the proof-of-concept that the use of a shotgun NGS metagenomics ED assay is a feasible and dependable approach for microbiome characterization and pathogen identification in laboratory animals. In its daily application, results suggest the usefulness of the test in SPF facilities (Miller and Brielmeier, 2018; Miller et al., 2016) where pathogenic micro-organisms are expected to be absent. mNGS analysis of ED filters allows the analysis of multiple cages, reducing the number of tests required for pathogen detection and corresponding costs, and avoiding the use of sentinel mice.

**Additional File 1. Normalized data from NGS analyses (non SPF)**

The different lines represent the different species identified in the non SPF facility with the normalized counts. For each species there are 8 columns that describe the taxonomy (superkingdom. phylum. class. order. family. genus. species. Species ID). The following columns identify the reads of each sample. The multiplier factor for normalizing stool samples on 1 million was variable, with an average of 2.5 (range between 1.4 and 3.9). This multiplication factor was applied to the filter count to allow comparison with the count number of fecal samples. P17-27: fecal samples collected from 11 non-SPF cages (in each cage mice n = 5); F-31: ED TEST filter; F-30: ED animal-free filter.

**Additional File 2. Normalized data form NGS analyses (SPF).**

The different lines represent the different species identified in the SPF facility with their respective counts. For each species there are 8 columns that describe the taxonomy (superkingdom. phylum. class. order. family. genus. species. species ID). The following columns identify the reads of each sample. R20/22 to R24/22: fecal sample collected from 5 SPF cages (mice n = 5 in each cage); F-26: SPF ED filter.

**Additional File 3. Normalized data from NGS analyses**.

The different lines represent the different species identified from pulverized clean chow and bedding DNA with their respective counts (Scavizzi et al., 2021). For each species there are 8 columns that describe the taxonomy (superkingdom. phylum. class. order. family. genus. species. species ID). The following columns identify the reads of each sample. Clean chow and bedding: DNA extracted from pulverized sample of chow and bedding taken from a microisolator cage (no animals) placed in the non SPF IVC rack for 4 weeks.

**Additional File. 4 Microbial families in SPF mouse fecal samples and in SPF filter.**

Column “family” lists the different microbial families identified by mNGS; column “fecal pellets”: average percentage of microbial families in SPF fecal pellets; column “ED filter”: microbial families percentage in the SPF ED filter. The percentage of microbial families deriving from chow and bedding is also indicated.

## Electronic supplementary material

Below is the link to the electronic supplementary material.

Supplementary Material 1

Supplementary Material 2

Supplementary Material 3

Supplementary Material 4

## Data Availability

All data generated or analysed during this study are included in this published article and its supplementary information files. The dataset supporting the conclusion of this article is included within the article and its additional files.

## References

[CR1] Antonini M, Lo Conte M, Sorini C, Falcone M (2019). How the interplay between the Commensal Microbiota, Gut Barrier Integrity, and Mucosal Immunity regulates Brain Autoimmunity. Front Immunol.

[CR2] Arentsen T, Raith H, Qian Y, Forssberg H, Diaz Heijtz R (2015). Host microbiota modulates development of social preference in mice. Microb Ecol Health Dis.

[CR3] Arpaia N, Campbell C, Fan X, Dikiy S, van der Veeken J, deRoos P, Liu H, Cross JR, Pfeffer K, Coffer PJ, Rudensky AY (2013). Metabolites produced by commensal bacteria promote peripheral regulatory T-cell generation. Nature.

[CR4] Bauer BA, Besch-Williford C, Livingston RS, Crim MJ, Riley LK, Myles MH (2016). Influence of rack design and disease prevalence on detection of Rodent Pathogens in Exhaust debris samples from individually ventilated Caging Systems. J Am Association Lab Anim Science: JAALAS.

[CR5] Benga L, Sager M, Christensen H (2018). From the [Pasteurella] pneumotropica complex to Rodentibacter spp.: an update on [Pasteurella] pneumotropica. Vet Microbiol.

[CR6] Buchheister S, Bleich A (2021) Health Monitoring of Laboratory Rodent Colonies-Talking about (R)evolution. *Animals (Basel)* 11 10.3390/ani11051410 PMC8155880 34069175

[CR7] Caspani G, Swann J (2019). Small talk: microbial metabolites involved in the signaling from microbiota to brain. Curr Opin Pharmacol.

[CR8] Chiu CY (2013). Viral pathogen discovery. Curr Opin Microbiol.

[CR9] Compton SR (2020). PCR and RT-PCR in the diagnosis of Laboratory Animal Infections and in Health Monitoring. J Am Association Lab Anim Science: JAALAS.

[CR10] Compton SR, Homberger FR, MacArthur Clark J (2004). Microbiological monitoring in individually ventilated cage systems. Lab Anim.

[CR11] Compton SR, Homberger FR, Paturzo FX, Clark JM (2004). Efficacy of three microbiological monitoring methods in a ventilated cage rack. Comp Med.

[CR12] de Bruin WC, van de Ven EM, Hooijmans CR (2016). Efficacy of soiled bedding transfer for transmission of mouse and rat infections to sentinels: a systematic review. PLoS ONE.

[CR13] Dubelko AR, Zuwannin M, McIntee SC, Livingston RS, Foley PL (2018) PCR testing of Filter Material from IVC Lids for Microbial Monitoring of Mouse Colonies.Journal of the American Association for Laboratory Animal Science: JAALAS 10.30802/AALAS-JAALAS-18-000008 PMC6159679 30092857

[CR14] Fung TC, Olson CA, Hsiao EY (2017). Interactions between the microbiota, immune and nervous systems in health and disease. Nat Neurosci.

[CR15] Furusawa Y, Obata Y, Fukuda S, Endo TA, Nakato G, Takahashi D, Nakanishi Y, Uetake C, Kato K, Kato T, Takahashi M, Fukuda NN, Murakami S, Miyauchi E, Hino S, Atarashi K, Onawa S, Fujimura Y, Lockett T, Clarke JM, Topping DL, Tomita M, Hori S, Ohara O, Morita T, Koseki H, Kikuchi J, Honda K, Hase K, Ohno H (2013). Commensal microbe-derived butyrate induces the differentiation of colonic regulatory T cells. Nature.

[CR16] Gerwin PM, Ricart Arbona RJ, Riedel ER, Henderson KS, Lipman NS (2017). PCR testing of IVC Filter tops as a method for detecting Murine Pinworms and Fur Mites. J Am Association Lab Anim Science: JAALAS.

[CR17] Hanson WH, Taylor K, Taylor DK (2021). PCR testing of media placed in Soiled Bedding as a method for mouse colony Health Surveillance. J Am Association Lab Anim Science: JAALAS.

[CR18] Kapoor P, Hayes YO, Jarrell LT, Bellinger DA, Thomas RD, Lawson GW, Arkema JD, Fletcher CA, Nielsen JN (2017). Evaluation of Anthelmintic Resistance and Exhaust Air Dust PCR as a Diagnostic Tool in mice enzootically infected with Aspiculuris tetraptera. J Am Association Lab Anim Science: JAALAS.

[CR19] Kau AL, Ahern PP, Griffin NW, Goodman AL, Gordon JI (2011). Human nutrition, the gut microbiome and the immune system. Nature.

[CR20] Kimie Niimi SM, Norihisa Sako K, Miyata T, Yoshimoto B, Bilecki KS, Henderson, Takahashi E (2018). The sentinel TM EADR program can detect more microorganisms than bedding sentinel animals. Jpn J Vet Res.

[CR21] Korner C, Miller M, Brielmeier M (2019). Detection of murine astrovirus and myocoptes musculinus in individually ventilated caging systems: investigations to expose suitable detection methods for routine hygienic monitoring. PLoS ONE.

[CR22] Langmead B, Salzberg SL (2012). Fast gapped-read alignment with Bowtie 2. Nat Methods.

[CR23] Lazar V, Ditu LM, Pircalabioru GG, Gheorghe I, Curutiu C, Holban AM, Picu A, Petcu L, Chifiriuc MC (2018). Aspects of gut microbiota and Immune System interactions in infectious Diseases, Immunopathology, and Cancer. Front Immunol.

[CR24] Levy M, Blacher E, Elinav E (2017). Microbiome, metabolites and host immunity. Curr Opin Microbiol.

[CR25] Ley RE, Turnbaugh PJ, Klein S, Gordon JI (2006). Microbial ecology: human gut microbes associated with obesity. Nature.

[CR26] Li H, Handsaker B, Wysoker A, Fennell T, Ruan J, Homer N, Marth G, Abecasis G, Durbin R (2009). The sequence Alignment/Map format and SAMtools. Bioinformatics.

[CR27] Lipman NS, Homberger FR (2003). Rodent quality assurance testing: use of sentinel animal systems. Lab Anim.

[CR28] Liu P, Peng G, Zhang N, Wang B, Luo B (2019). Crosstalk between the gut microbiota and the brain: an update on neuroimaging findings. Front Neurol.

[CR29] Lozupone CA, Stombaugh JI, Gordon JI, Jansson JK, Knight R (2012). Diversity, stability and resilience of the human gut microbiota. Nature.

[CR30] Lu J, Breitwieser FP, Thielen P, Salzberg SL (2017). Bracken: estimating species abundance in metagenomics data. Peer J Computer Science.

[CR31] Luchins KR, Bowers CJ, Mailhiot D, Theriault BR, Langan GP (2020). Cost comparison of Rodent Soiled Bedding Sentinel and Exhaust Air Dust Health-Monitoring Programs. J Am Association Lab Anim Science: JAALAS.

[CR32] Luchins KR, Mailhiot D, Theriault BR, Langan GP (2020). Detection of Lactate Dehydrogenase Elevating Virus in a mouse Vivarium using an Exhaust Air Dust Health Monitoring Program. J Am Association Lab Anim Science: JAALAS.

[CR33] Mahabir E, Durand S, Henderson KS, Hardy P (2019). Comparison of two prevalent individually ventilated caging systems for detection of murine infectious agents via exhaust air particles. Lab Anim.

[CR34] Mailhiot D, Ostdiek AM, Luchins KR, Bowers CJ, Theriault BR, Langan GP (2020). Comparing Mouse Health Monitoring between soiled-bedding Sentinel and Exhaust Air Dust Surveillance Programs. J Am Association Lab Anim Science: JAALAS.

[CR35] Manuel CA, Pugazhenthi U, Leszczynski JK (2016). Surveillance of a ventilated rack System for Corynebacterium bovis by Sampling Exhaust-Air Manifolds. J Am Association Lab Anim Science: JAALAS.

[CR36] Manuel CA, Pugazhenthi U, Spiegel SP, Leszczynski JK (2017). Detection and elimination of Corynebacterium bovis from Barrier rooms by using an environmental Sampling Surveillance Program. J Am Association Lab Anim Science: JAALAS.

[CR37] Matson V, Chervin CS, Gajewski TF (2021). Cancer and the Microbiome-Influence of the commensal microbiota on Cancer, Immune responses, and Immunotherapy. Gastroenterology.

[CR38] Miller M, Brielmeier M (2018). Environmental samples make soiled bedding sentinels dispensable for hygienic monitoring of IVC-reared mouse colonies. Lab Anim.

[CR39] Miller M, Ritter B, Zorn J, Brielmeier M (2016). Exhaust Air Dust Monitoring is Superior to Soiled Bedding Sentinels for the detection of Pasteurella pneumotropica in individually ventilated Cage Systems. J Am Association Lab Anim Science: JAALAS.

[CR40] Needham BD, Funabashi M, Adame MD, Wang Z, Boktor JC, Haney J, Wu WL, Rabut C, Ladinsky MS, Hwang SJ, Guo Y, Zhu Q, Griffiths JA, Knight R, Bjorkman PJ, Shapiro MG, Geschwind DH, Holschneider DP, Fischbach MA, Mazmanian SK (2022). A gut-derived metabolite alters brain activity and anxiety behaviour in mice. Nature.

[CR41] O’Connell KA, Tigyi GJ, Livingston RS, Johnson DL, Hamilton DJ (2021). Evaluation of In-cage Filter Paper as a replacement for Sentinel mice in the detection of murine pathogens. J Am Association Lab Anim Science: JAALAS.

[CR42] Pernigoni N, Zagato E, Calcinotto A, Troiani M, Mestre RP, Cali B, Attanasio G, Troisi J, Minini M, Mosole S, Revandkar A, Pasquini E, Elia AR, Bossi D, Rinaldi A, Rescigno P, Flohr P, Hunt J, Neeb A, Buroni L, Guo C, Welti J, Ferrari M, Grioni M, Gauthier J, Gharaibeh RZ, Palmisano A, Lucchini GM, D’Antonio E, Merler S, Bolis M, Grassi F, Esposito A, Bellone M, Briganti A, Rescigno M, Theurillat JP, Jobin C, Gillessen S, de Bono J, Alimonti A (2021). Commensal bacteria promote endocrine resistance in prostate cancer through androgen biosynthesis. Science.

[CR43] Pettan-Brewer C, Trost RJ, Maggio-Price L, Seamons A, Dowling SC (2020). Adoption of Exhaust Air Dust Testing in SPF Rodent Facilities. J Am Association Lab Anim Science: JAALAS.

[CR44] Salipante SJ, Hoogestraat DR, Abbott AN, SenGupta DJ, Cummings LA, Butler-Wu SM, Stephens K, Cookson BT, Hoffman NG (2014). Coinfection of Fusobacterium nucleatum and Actinomyces israelii in mastoiditis diagnosed by next-generation DNA sequencing. J Clin Microbiol.

[CR45] Salipante SJ, SenGupta DJ, Cummings LA, Land TA, Hoogestraat DR, Cookson BT (2015). Application of whole-genome sequencing for bacterial strain typing in molecular epidemiology. J Clin Microbiol.

[CR46] Sampson TR, Mazmanian SK (2015). Control of brain development, function, and behavior by the microbiome. Cell Host Microbe.

[CR47] Scavizzi F, Bassi C, Lupini L, Guerriero P, Raspa M, Sabbioni S (2021). A comprehensive approach for microbiota and health monitoring in mouse colonies using metagenomic shotgun sequencing. Anim Microbiome.

[CR48] Schmieder R, Edwards R (2011). Quality control and preprocessing of metagenomic datasets. Bioinformatics.

[CR49] Sharon G, Sampson TR, Geschwind DH, Mazmanian SK (2016). The Central Nervous System and the gut Microbiome. Cell.

[CR50] Smith PM, Howitt MR, Panikov N, Michaud M, Gallini CA, Bohlooly YM, Glickman JN, Garrett WS (2013). The microbial metabolites, short-chain fatty acids, regulate colonic Treg cell homeostasis. Science.

[CR51] Tang TWH, Chen HC, Chen CY, Yen CYT, Lin CJ, Prajnamitra RP, Chen LL, Ruan SC, Lin JH, Lin PJ, Lu HH, Kuo CW, Chang CM, Hall AD, Vivas EI, Shui JW, Chen P, Hacker TA, Rey FE, Kamp TJ, Hsieh PCH (2019). Loss of Gut Microbiota alters Immune System Composition and cripples Postinfarction Cardiac Repair. Circulation.

[CR52] Taylor NS, Xu S, Nambiar P, Dewhirst FE, Fox JG (2007). Enterohepatic Helicobacter species are prevalent in mice from commercial and academic institutions in Asia, Europe, and North America. J Clin Microbiol.

[CR53] Vicentini FA, Keenan CM, Wallace LE, Woods C, Cavin JB, Flockton AR, Macklin WB, Belkind-Gerson J, Hirota SA, Sharkey KA (2021). Intestinal microbiota shapes gut physiology and regulates enteric neurons and glia. Microbiome.

[CR54] WMS, Russel RB (1959) The principles of humane experimental technique

[CR55] Wood DE, Salzberg SL (2014). Kraken: ultrafast metagenomic sequence classification using exact alignments. Genome Biol.

[CR56] Yu F, Han W, Zhan G, Li S, Jiang X, Wang L, Xiang S, Zhu B, Yang L, Luo A, Hua F, Yang C (2019). Abnormal gut microbiota composition contributes to the development of type 2 diabetes mellitus in db/db mice. Aging.

[CR57] Zorn J, Ritter B, Miller M, Kraus M, Northrup E, Brielmeier M (2017). Murine norovirus detection in the exhaust air of IVCs is more sensitive than serological analysis of soiled bedding sentinels. Lab Anim.

